# Public attitudes towards immigration, news and social media exposure, and political attitudes from a cross-cultural perspective: Data from seven European countries, the United States, and Colombia

**DOI:** 10.1016/j.dib.2021.107548

**Published:** 2021-11-07

**Authors:** David De Coninck, Maria Duque, Seth J. Schwartz, Leen d'Haenens

**Affiliations:** a Centre for Sociological Research, KU Leuven, Belgium; b Department of Kinesiology and Health Education, University of Texas at Austin, United States; c Institute for Media Studies, KU Leuven, Belgium

**Keywords:** News media consumption, Social media use, Anti-immigrant attitudes, Political attitudes, Threat perceptions

## Abstract

The data presented in this article provide the opportunity to comparatively analyse anti-immigrant and anti-refugee attitudes, news and social media consumption, and political attitudes (e.g., social dominance orientation, right-wing authoritarianism) of the adult population in seven European countries (Austria, Belgium, Germany, Hungary, Italy, Spain, Sweden), the United States, and Colombia in 2021 (*N* = 13,645). These countries were selected for their variety in national characteristics: coastal and non-coastal border countries, large and small economies, countries with major and minor political influence, and countries with varying degrees of popularity as asylum-seeker destinations. We conducted an online survey which – amongst others – included questions on socio-demographic characteristics, attitudinal indicators, and information on news and social media consumption. These data can be of interest for migration researchers and/or media scholars who want to explore (comparative) dynamics of outgroup attitudes, threat perceptions, and/or news and social media consumption, and for policy makers who seek to influence public attitudes towards immigration and migrants.

## Specifications Table

**Table:** 

Subject	Sociology and Political Science; Media Studies
Specific subject area	Attitudes towards immigration; News media consumption; Social media use; Political attitudes
Type of data	Table
How data were acquired	Online survey among the adult population in Austria, Belgium, Germany, Hungary, Italy, Spain, Sweden, the United States, and Colombia
Data format	Raw
Parameters for data collection	Being over the age of 24 and under the age of 66 (for the European countries) and being over the age of 17 and under the age of 66 (for the United States and Colombia), and residing in one of these countries at the time of the fieldwork (May through June of 2021).
Description of data collection	We collected the data in cooperation with a Belgian polling agency (and its affiliated agencies) and selected the methodology for its cost-effectiveness in cross-country research. Bilendi, the polling agency we worked with, has a strong presence in all countries, which facilitated fieldwork. Respondents received an e-mail asking them to participate in a survey without specifying the subject matter, which was essential to avoid priming. Three weeks of fieldwork in May and June of 2021 resulted in a dataset of 13,645 respondents (about 1500 per country). Sample weights were applied to ensure representativeness of the sample for gender and age in each country. The cooperation rate ranged from 12% to 31%.
Data source location	Institution: KU Leuven/University of Texas at Austin City/Town/Region: Leuven/Austin, TX Country: Belgium/United States
Data accessibility	Repository name: Mendeley Data Data identification number: http://dx.doi.org/10.17632/8mgpmdstp2.2 Direct URL to data: https://data.mendeley.com/datasets/8mgpmdstp2.2

## Value of the Data

• The data presented can contribute to a better understanding of the relationship between individual attitudes towards outgroups (immigrants, refugees, Muslims, Hispanics…), the consumption of and trust in specific news and social media, and socio-political attitudes (authoritarianism, social dominance) in Austria, Belgium, Germany, Hungary, Italy, Spain, Sweden, the U.S., and Colombia.
• Researchers in migration studies can benefit from these data because they highlight how attitudes differ according to specific groups of newcomers (immigrants, refugees, Muslims, Hispanics), using relevant socio-political attitudes such as right-wing authoritarianism, social dominance orientation, political efficacy.
• Researchers of media effects can benefit from the detailed measurements of news media consumption and trust, as differences between public and commercial broadcasters and quality and popular newspapers are highlighted. Furthermore, the detailed measurement of social media activity allows for a better understanding of the relationship between this media use and individual attitudes.
• The data presented are unique: although the field of public attitudes towards immigration has grown considerably, there is little research that combines indicators on intergroup attitudes with measures on (news) media in this wide range of countries. This allows for new insights in a field which is rapidly evolving, and where researchers and policy makers alike are looking for new knowledge on drivers of attitudes towards migration.

## 1 Data Description

The data presented in this article were collected in the context of two H2020 research projects: ‘Enhanced migration measures from a multidimensional perspective’ (HumMingBird) and ‘Crises as opportunities: Towards a level telling field on migration and a new narrative of successful integration’ (OPPORTUNITIES). The current survey was fielded to investigate the dynamic interplay between media representations of different migrant groups and the governmental and societal (re)actions to immigration. With these data, we provide more insight into these societal reactions by investigating attitudes rooted in values and worldviews. Through an online survey, we collected quantitative data on attitudes towards immigrants, refugees, Muslims, Hispanics…, news media consumption, trust in news media and societal institutions, frequency and valence of intergroup contact, realistic and symbolic intergroup threat, right-wing authoritarianism, social dominance orientation, political efficacy, personality characteristics, perceived COVID-threat, and socio-demographic characteristics for the adult population aged 25 to 65 in seven European countries: Austria, Belgium, Germany, Hungary, Italy, Spain, Sweden. In the United States and Colombia, ages ranged from 18 to 65. The survey in the United States and Colombia was identical to the one in the European countries, although a few extra questions regarding COVID-19 and some region-specific migrant groups (e.g. Venezuelans) were added. We collected the data in cooperation with Bilendi, a Belgian polling agency, and selected the methodology for its cost-effectiveness in cross-country research. Respondents received an e-mail asking them to participate in a survey without specifying the subject matter, which was essential to avoid priming. Three weeks of fieldwork in May and June of 2021 resulted in a dataset of 13,645 respondents (a little over 1500 per country). Sample weights are included in the dataset and can be applied to ensure that the sample is representative for gender and age in each country. The cooperation rate ranged between 12% and 31%, in line with similar online data collections [1]. While Table 1 shows the distribution of respondents by several socio-demographic characteristics, Table 2 presents mean scores of selected indicators on attitudes, political attitudes, news media consumption and trust, perceived threat, and intergroup contact.

**Table 1:** Descriptive overview of the sample (*N* = 13,645).

	Austria	Belgium	Colombia	Germany	Hungary	Italy	Spain	Sweden	U.S.
In %									
**Gender**									
Male	50.6	48.1	43.3	49.0	46.5	48.8	50.5	50.3	51.8
Female	49.4	51.9	56.7	51.0	53.5	51.2	49.5	49.7	48.2
**Age**									
Under 30 years	12.2	9.0	33.8	9.7	8.9	6.6	8.7	10.1	13.1
Between 30 and 45 years	42.1	39.0	41.3	41.2	45.8	40.6	44.6	41.9	43.3
Between 45 and 60 years	35.7	40.4	19.8	38.4	34.4	42.2	37.2	38.7	22.0
Over 60 years	10.0	11.6	5.1	10.7	10.9	10.6	9.5	9.4	21.6
**Religious denomination**									
Roman Catholic	57.9	45.3	61.8	27.2	48.3	72.4	51.7	6.5	34.8
Protestant	5.7	2.1	7.9	27.9	6.9	1.0	1.2	27.4	22.3
Muslim	2.5	1.3	0.4	2.0	0.2	0.7	0.4	3.8	6.1
Jewish	0.5	0.4	0.9	0.5	0.4	0.1	0.1	0.2	5.1
Agnostic/Atheist	25.7	46.5	4.9	36.8	24.9	20.1	39.2	51.5	10.0
Other	7.7	4.5	24.0	5.6	19.2	5.6	7.1	10.7	21.6
**Full-time job**	56.8	63.5	45.9	58.6	65.6	55.5	62.2	63.7	59.7
**Born in country of residence**	87.9	95.5	92.0	93.9	97.4	96.0	91.5	86.2	93.9
**Type of region of residence**									
In the countryside	8.0	4.8	9.8	3.1	1.2	5.2	4.6	8.0	11.1
Small town	25.9	35.1	0.3	20.7	15.5	19.4	1.4	10.5	15.8
Small city	27.2	25.0	23.3	39.1	31.2	44.1	39.0	27.8	10.8
Suburbs or outskirts of a big city	10.4	17.8	5.5	12.4	13.1	9.9	11.6	20.8	29.5
Big city	28.5	14.3	61.1	24.7	39.0	21.4	43.3	32.9	32.8

In mean scores (standard error between brackets)									

**Educational attainment**	3.37 (0.85)	3.81 (1.01)	3.09 (1.15)	3.24 (1.05)	3.52 (0.76)	3.53 (1.00)	3.56 (1.12)	3.53 (0.84)	4.09 (1.30)
**Political ideology**	4.79 (2.11)	5.32 (2.21)	5.10 (1.81)	4.77 (1.97)	5.39 (2.35)	5.21 (2.53)	4.47 (2.24)	5.37 (2.59)	6.46 (2.87)
Financial deprivation*	3.65 (1.26)	3.88 (1.15)	2.95 (1.15)	3.80 (1.20)	3.27 (1.10)	3.38 (1.14)	3.52 (1.22)	3.96 (1.34)	4.85 (1.77)

*N*	*1,520*	*1,505*	*1,543*	*1,521*	*1,514*	*1,510*	*1,512*	*1,517*	*1,503*
*Cooperation rate (in %)*	*23*	*22*	*19*	*22*	*31*	*19*	*16*	*12*	*17*

Note: ^⁎^ European countries and Colombia used a six-point scale, the U.S. used a seven-point scale. High scores indicate low financial deprivation.

**Table 2:** Mean scores of selected indicators on attitudes, news media consumption, threat, and intergroup contact.

	Austria	Belgium	Colombia	Germany	Hungary	Italy	Spain	Sweden	U.S.
In mean scores (standard error between brackets)									

**Feeling thermometers**									
Immigrants	5.20 (2.44)	5.16 (2.50)	6.52 (2.33)	5.42 (2.41)	4.07 (2.48)	5.59 (2.59)	6.17 (2.45)	5.07 (2.73)	6.81 (2.67)
Refugees	5.04 (2.44)	5.40 (2.46)	6.55 (2.13)	5.29 (2.37)	4.42 (2.42)	6.07 (2.55)	6.62 (2.32)	5.07 (2.70)	6.70 (2.63)
Muslims	4.13 (2.58)	4.91 (2.71)	-	4.55 (2.55)	3.61 (2.34)	4.71 (2.63)	4.83 (4.71)	3.98 (2.89)	-
Hispanics	-	-	-	-	-	-	-	-	6.87 (2.60)
Venezuelans	-	-	5.64 (2.52)	-	-	-	-	-	-
**Public television consumption**	3.96 (2.20)	4.46 (2.13)	4.53 (2.14)	4.47 (2.09)	3.01 (2.06)	4.51 (2.03)	4.49 (2.15)	4.11 (2.01)	3.39 (2.31)
**Commercial television consumption**	3.89 (1.94)	3.83 (2.17)	4.65 (2.30)	3.88 (2.05)	3.93 (2.15)	4.38 (2.03)	4.94 (2.03)	4.13 (1.97)	-
CNN (U.S. only)	-	-	-	-	-	-	-	-	3.67 (2.35)
Fox News (U.S. only)	-	-	-	-	-	-	-	-	3.69 (2.32)
MSNBC (U.S. only)	-	-	-	-	-	-	-	-	3.36 (2.28)
**Quality newspaper consumption**	2.16 (1.37)	2.09 (1.42)	2.58 (1.50)	1.82 (1.26)	1.64 (1.11)	2.37 (1.46)	2.05 (1.39)	2.15 (1.39)	-
**Popular newspaper consumption**	2.43 (1.32)	2.22 (1.25)	2.37 (1.39)	1.70 (1.17)	1.79 (1.18)	2.04 (1.60)	2.38 (1.42)	3.37 (1.93)	-
Newspaper consumption (U.S. only)	-	-	-	-	-	-	-	-	3.09 (2.15)
**Quality news website consumption**	2.35 (1.38)	2.59 (1.53)	2.68 (1.55)	2.23 (1.38)	2.75 (1.52)	2.59 (1.52)	1.80 (1.17)	2.72 (1.51)	3.16 (2.17)
**Popular news website consumption**	2.00 (1.23)	2.47 (1.36)	3.31 (1.81)	2.18 (1.51)	2.74 (1.39)	2.80 (1.59)	1.92 (1.37)	3.69 (1.95)	3.27 (2.33)
**Social media use**									
Stay informed about current events	4.15 (1.93)	3.53 (1.90)	5.22 (1.81)	4.03 (1.95)	4.31 (1.85)	4.12 (1.87)	4.30 (1.71)	3.95 (1.79)	4.35 (2.01)
Stay informed about local community	3.70 (1.92)	3.85 (1.72)	5.08 (1.82)	3.86 (1.96)	4.21 (1.77)	4.44 (1.77)	4.32 (1.74)	3.92 (1.72)	4.51 (1.96)
Get news from mainstream media	3.62 (1.96)	3.46 (1.88)	4.85 (1.91)	3.56 (1.94)	3.89 (1.91)	4.27 (1.83)	3.98 (1.77)	3.65 (1.83)	4.21 (2.12)
Get news through friends	3.90 (1.91)	3.96 (1.79)	4.73 (1.89)	3.93 (1.93)	4.10 (1.83)	4.05 (1.80)	4.08 (1.71)	3.82 (1.73)	4.29 (2.07)
Meet new people	2.92 (1.78)	2.64 (1.65)	4.13 (1.98)	3.09 (1.84)	3.48 (1.80)	3.51 (1.82)	3.62 (1.85)	3.20 (1.74)	4.16 (2.13)
**Contact with EU migrants**	3.05 (1.12)	2.67 (1.16)	-	2.91 (1.09)	1.96 (0.98)	2.71 (1.12)	2.85 (1.07)	3.40 (1.05)	-
**Contact with non-EU migrants**	2.92 (1.13)	2.63 (1.17)	-	2.84 (1.10)	1.85 (0.95)	2.82 (1.15)	2.97 (1.10)	3.44 (1.06)	-
**Intergroup threat**									
Crime	6.97 (2.07)	6.34 (1.86)	7.44 (1.96)	6.72 (2.04)	5.97 (1.82)	6.53 (2.00)	5.96 (1.96)	7.55 (2.22)	6.60 (2.24)
Jobs	4.91 (2.18)	4.96 (2.04)	4.74 (2.71)	5.11 (2.22)	4.43 (2.17)	4.97 (2.36)	5.22 (2.31)	5.35 (2.49)	6.07 (2.72)
Taxes	4.04 (2.52)	4.32 (2.30)	4.37 (2.35)	4.42 (2.48)	4.17 (2.09)	5.01 (2.40)	4.90 (2.42)	3.96 (2.68)	5.91 (2.88)
Economy	4.81 (2.49)	4.72 (2.34)	4.58 (2.47)	5.34 (2.46)	4.22 (2.35)	5.12 (2.48)	5.40 (2.45)	4.28 (2.92)	6.27 (2.80)
Culture	4.47 (2.77)	5.13 (2.54)	5.06 (2.28)	5.13 (2.70)	4.46 (2.50)	5.46 (2.60)	5.75 (2.49)	5.13 (2.87)	2.87 (2.70)
Values	5.10 (2.79)	4.66 (2.50)	5.30 (2.29)	5.16 (2.60)	4.03 (2.38)	4.89 (2.58)	5.41 (2.47)	4.27 (2.66)	2.66 (2.67)

*N*	*1,520*	*1,505*	*1,543*	*1,521*	*1,514*	*1,510*	*1,512*	*1,517*	*1,503*

Note: Scores on the feeling thermometer were recoded to a 10-point scale.

## 2 Experimental Design, Materials and Methods

Despite the growing attention to the role of news media as a contextual driver in the attitude formation to migration, many national and international data sources continue to exhibit a variety of shortcomings (limited to a single country, lack of detail in media consumption measurement, vague attitude measures). This dataset aims to remedy some of these shortcomings, for example by including more information on news media exposure and news media trust (among others) alongside variables measuring attitudes towards different outgroups. To summarize, we developed an online public opinion survey that was fielded among Austrian, Belgian, Colombian, German, Hungarian, Italian, Spanish, Swedish, and U.S. residents aged 25 (or 18 for Colombia and U.S.) to 65, representative for gender and age. The survey was distributed by Bilendi, a market research and online polling agency with opt-in panels, active in all countries included in the dataset. The fieldwork included the use of incentives to maximize the response rate. Participants who completed the survey received a number of points that they were able to save in exchange for coupons. The survey we developed consists of seven themes: socio-demographic characteristics, political attitudes, media- and news consumption, trust in media and societal institutions, attitudes towards and contact with migrants, psychological indicators, and COVID-19-indicators. In what follows, we highlight several measures. All data were processed and cleaned through SPSS Version 26. Below, we provide a brief overview of some of the variables in the dataset.

### 2.1 Public attitudes towards outgroups

To assess sentiments towards outgroups in each country, we presented a feeling thermometer question. Respondents were asked to indicate how they felt towards immigrants, refugees, … with a score of 0 representing very cold or negative feelings while a score of 10 indicates very warm or positive feelings. In order to ensure that all respondents had a uniform understanding of the individuals that we considered to be an immigrant or a refugee, we presented the UN definition of these groups:

 “An immigrant should be understood as covering all cases where the decision to migrate is taken freely by the individual concerned, for reasons of 'personal convenience' and without intervention of an external compelling reason (e.g., war, natural disaster, …)” [2].
 “A refugee is someone who has been forced to flee his or her country because of persecution, war, or violence. A refugee has a well-founded fear of persecution for reasons of race, religion, nationality, political opinion or membership in a particular social group” [3].

We clearly highlighted these two definitions so that respondents would be able to distinguish between immigrants and refugees and provide a reliable measurement of attitudes for each group. Furthermore, we also asked about feelings towards Muslims (not in U.S. and Colombia), Hispanics (U.S. only), and Venezuelans (Colombia only).

### 2.2 News consumption

Respondents were asked about their news consumption patterns via traditional broadcast, press, and online news platforms during the past month, with answer categories ranging from 1 (never) to 7 (every day). Both television and radio consumption were split into two groups: public and commercial broadcasters. For the newspaper and online news consumption, the most commonly read newspapers and commonly visited webpages in each country were included separately. This selection of newspapers was based on information concerning the circulation of newspapers in each country. Online and social media were also included. For online media, most of the websites of the leading newspapers were selected, with some additional online-only news outlets.

To have an indication of social media use, we used a previously tested measure by Diehl et al. [4]. The question asked is to what extent respondents have used social media to…: (1) stay informed about current events and public affairs, (2) get news about their local communities, (3) get news about current events from mainstream media (e.g., professional news services), (4) get news about current events from friends, and (5) to meet new people who share their interests. Answer categories range from 1 (never) to 7 (all the time).

### 2.3 Perceived intergroup threat and intergroup contact

To measure perceived intergroup threat, we included two main types of threat. A first set of indicators assesses economic (or realistic) threat. This was measured by four statements/questions: ‘*Have [your country]’s crime problems increased or decreased by refugees coming to live here?*’, ‘*Would you say that refugees who come to live here generally take jobs away from workers in [your country], or generally help to create new jobs?*’, ‘*Most refugees who come to live here work and pay taxes. They also use health and welfare services. On balance, do you think refugees who come here take out more than they put in or put in more than they take out?*’, and *‘Would you say it is generally bad or good for [your country]'s economy that refugees come to live here from other countries?*’. Two items were presented to assess symbolic (or cultural) threat: ‘*Would you say that [your country]’s cultural life is generally undermined or enriched by refugees coming to live here from other countries?*’ and ‘*Generally speaking, values and beliefs of refugees are not compatible with those of [your country]*’. Both types of threat were measured on an 11-point scale, with the high end of the scale signifying a low degree of threat. These indicators on economic and cultural threat stem from the rotating module on immigration in Round 1 and Round 7 of the European Social Survey [5].

Direct intergroup contact was measured by asking whether respondents have any contact (1 = never, 5 = every day) and/or friendships (1 = none, 5 = all of them) with EU-migrants or with non-EU migrants. In the U.S., alternate categories of migrants were presented: migrants from Europe/Central or South America, Africa or Arab countries/Asia/Australia or Antarctica. In Colombia, the categories were: immigrants from Venezuela/other parts of Latin America or the Caribbean/North America/other parts of the world.

## Ethics Statement

Informed consent was obtained from all participants prior to participation in the study. Ethical approval was obtained from the Social and Societal Ethics Committee of KU Leuven (G-2020-2590).

## CRediT Author Statement

**David De Coninck:** Conceptualization, Methodology, Software; **Maria Duque:** Conceptualization, Methodology; **Seth J. Schwartz:** Conceptualization, Supervision; **Leen d'Haenens:** Supervision, Writing – review & editing.

## Declaration of Competing Interest

The authors declare that they have no known competing financial interests or personal relationships which have or could be perceived to have influenced the work reported in this article.
